# Application value of ultrasonic contrast imaging and ultrasonic parameters in post-transplant renal surgery

**DOI:** 10.3389/fmed.2024.1397884

**Published:** 2024-08-27

**Authors:** Xinwei Liu, Dikuan Liu, Meizhen Long, Feng Chen

**Affiliations:** The Affiliated Yiyang Central Hospital, Hunan University of Chinese Medicine, Yiyang, China

**Keywords:** acute rejection, delayed renal function recovery, renal transplantation, ultrasonic contrast imaging, VUEBOX quantitative analysis

## Abstract

**Objective:**

Utilize VUEBOX quantitative analysis software to perform quantitative analysis dynamic ultrasound contrast images of post-transplant renal patients were assessed quantitatively five parameters of ultrasonic contrast and two-dimensional ultrasound are examined to explore their six value in Diagnosing Renal Graft Dysfunction.

**Methods:**

A retrospective analysis was conducted on 73 post-transplant renal patients who underwent ultrasound contrast examinations at Yiyang Central Hospital from July 2022 to December 2023, They were diagnosed clinically and pathologically. Based on pathological and clinical diagnostic results, the patients were divided into three groups: 47 cases in the stable renal function group, 18 cases in the acute rejection (AR) group, and 8 cases in the delayed graft function (DGF) group. All patients underwent routine ultrasound and ultrasound contrast examinations post-transplantation. By comprehensively assessing renal function test results, clinical course, and pathological findings, differences in ultrasonic contrast quantitative parameters were analyzed. Additionally, ROC curves were constructed to evaluate the diagnostic efficacy of ultrasound contrast in discriminating between transplant renal rejection reactions and delayed renal function recovery.

**Results:**

Statistically significant differences in characteristics, such as renal segmental artery resistance index, were observed among the stable renal function group, AR group, and DGF group (all *P* < 0.05), while peak systolic velocity showed no statistical significance (*P* > 0.05). Differences in cortical time to peak (TTP), medullary time to peak(TTP), main renal artery rise time (RT), main renal artery(TTP), and main renal artery fall time (FT) were statistically significant among the stable renal function group, AR group, and DGF group (*P* < 0.05). ROC curve analysis demonstrated that the accuracy of quantitative parameters for the DGF group and AR group was as follows: Renal artery TTP = Renal artery RT > Renal artery FT > Medulla TTP > Cortex TTP (with respective area under the curve values of 0.828, 0.828, 0.758, 0.742, 0.719). Among these, Renal artery TTP and Renal artery RT exhibited larger AUC values, with sensitivities of 87.5% each and specificities of 81.2 and 87.5%, respectively.

**Conclusion:**

There are discernible differences in VUEBOX quantitative parameters between post-transplant AR and DGF cases, thereby providing imaging references for diagnosing of acute rejection and functional impairment following renal transplantation.

## 1 Introduction

Renal transplantation currently represents the most effective and beneficial therapeutic intervention for end-stage renal disease. With the continuous emergence of novel immunosuppressive drugs and the ongoing refinement of postoperative immunosuppressive treatment regimens, the incidence of acute rejection reactions and postoperative delayed graft function (DGF) in renal transplantation has historically decreased, leading to prolonged graft survival times. Nonetheless, acute rejection reactions and postoperative DGF persist as common and serious complications among renal transplant recipients. Clinical diagnosis of acute rejection reactions and DGF in transplants typically relies on pathology as the “gold standard,” with clinical presentation serving as a secondary criterion for diagnosis. However, percutaneous biopsy as an invasive diagnostic measure carries numerous drawbacks, including risks of bleeding, infection, renal parenchymal injury, and the potential for false-negative results due to the uneven distribution of lesion sites. Therefore, there is an urgent need for a non-invasive and efficient method to assist in the clinical diagnosis and treatment of acute rejection reactions and DGF.

Ultrasonic technology possesses the advantages of convenience, non-invasiveness, and radiation-free imaging. It enables the observation of renal structure and hemodynamic changes, making it one of the most commonly utilized methods for monitoring renal graft function. With the continuous advancement of ultrasonic technology, techniques such as ultrasonic contrast imaging have gradually found application in clinical practice, providing robust support for disease diagnosis and treatment. Ultrasonic contrast imaging utilizes microbubble contrast agents (SonoVue, Bracco, Italy), which are not metabolized by the kidneys but rather excreted via the respiratory system, rendering them non-toxic to transplanted kidneys. Extensive animal experiments and clinical trials have verified their safety (1, 2). The 2017 European Federation of Societies for Ultrasound in Medicine and Biology strongly advocated for the use of ultrasonic contrast imaging technology in diagnosing ischemia and microvascular complications (such as inflammation, thrombosis, etc.) following renal transplantation (3, 4). Ultrasonic contrast imaging holds clear advantages in evaluating organ microcirculation and blood perfusion. However, current research on the application of ultrasonic contrast imaging in DGF and acute rejection reactions is relatively limited, with inconsistent findings regarding its efficacy in assessing delayed graft function recovery and acute rejection reactions following renal transplantation (5). Therefore, this study aims to utilize the external perfusion software VUEBOX to delve into the clinical value of ultrasonic contrast imaging quantitative parameters in evaluating acute rejection reactions and delayed graft function in renal transplantation.

The main contributions of this study are as follows:

1. Based on CEUS, this study effectively and quantitatively demonstrates renal microvascular perfusion in AR patients, DGF patients, and normal control participants.
2. From the data sources, this study utilizes several CEUS parameters related to perfusion, such as TTP, which can serve as new markers for renal vascular perfusion.
3. The ROC curves constructed from the data obtained using VUEBOX quantitative analysis software provide clinicians with new insights for early differentiation between AR and DGF.

## 2 Materials and methods

Study Population: This study employed a retrospective analysis method. Inclusion criteria: all patients aged over 18 years who were admitted to or followed up at the renal transplant department of Yiyang Central Hospital from July 2023 to December 2023, and who underwent ultrasound and ultrasound contrast examinations. Exclusion criteria:

1. Patients with complications of the urinary system (such as significant renal hydronephrosis or effusion) and major vascular complications (such as renal artery stenosis and arterial/venous thrombosis);
2. Patients with contraindications to ultrasonic contrast agents, including those with a history of allergy, recent acute coronary syndrome, or clinically unstable heart disease (acute heart failure, NYHA functional class III/IV and severe arrhythmia), severe pulmonary arterial hypertension, pregnant or lactating patients;
3. Patients who did not consent to participate in the study.

Finally, 73 patients were included. Based on post-transplant graft function recovery and pathological results, renal transplant recipients were divided into the DGF group (*n* = 8), stable renal function group (*n* = 47), and AR group (*n* = 18). All patients provided informed consent.

Diagnostic Criteria for the DGF Group:

1. The need for dialysis therapy within the first week post-renal transplantation.
2. Early postoperative urine output < 1,200 mL/day.
3. Serum creatinine (SCr) concentration declining by < 10% per day in the first 3 days postoperatively or SCr not decreasing to 400 μmol/L within 1 week postoperatively.

Diagnostic Criteria for the Stable Renal Function Group:

1. Gradual normalization of urine output to 1,500–2,000 mL/day within 1 week post-renal transplantation.
2. Absence of signs such as fever, hypertension, enlargement of the transplanted kidney, and tenderness in the transplanted kidney area.

Clinical Major Diagnostic Criteria for Acute Rejection Reaction: Pathologically confirmed acute rejection reaction.

Clinical Secondary Criteria:

1. Unexplained decrease in urine output.
2. Unexplained rise in serum (increase of more than 30 mmol/L in 24 h).
3. Unexplained increase in urinary protein.
4. Significant enlargement of the transplanted kidney as indicated by renal ultrasound.
5. Unexplained low to moderate fever.
6. Pain the area of the kidney area.

## 3 Instruments and methods

The LOGIQ E11, GE, ultrasound system was utilized for the ultrasound examination. The conventional ultrasound used the C1-6 probe, while the ultrasound contrast employed the L2-9 probe, MI0.12-0.14. The examination was conducted by a physician with 5–10 years of experience in ultrasonic contrast imaging diagnosis, who documented the patient's gender and age. The patient was positioned supine, and the probe was gently placed over the transplanted kidney to clearly visualize the lesion and surrounding tissues. The two-dimensional ultrasound appearance of the lesion and color Doppler blood flow were carefully observed. SonoVue, a microbubble contrast agent manufactured by Bracco, was selected for ultrasonic contrast imaging. The microbubbles are phospholipid-coated sulfur hexafluoride with an average diameter of 2.5 μm, suspended in a 5 mL saline solution. The ultrasonic contrast mode was activated, and the contrast agent was injected while simultaneously starting the timer. Dynamic images were stored for 40–60 s. The transplanted kidney was observed twice consecutively, with 0.8 ml of UCA injected each time. During the first observation, the focus was on the renal cortex and medulla. After a 15-min interval, contrast agent was injected again to closely observe the renal artery of the transplanted kidney. The dynamic ultrasound contrast video was uploaded to the VUEBOX software in DICOM format for quantitative analysis (6); Using the VUEBOX quantitative analysis software, the region of interest (ROI) delineation was performed as follows (7, 8): ROI 1 was the area closest to the abdominal wall, comprising a portion of the cortex and medulla, with a minimum area of 0.1 cm^2^ for all four regions. ROI 2 represented the cortical region, ROI 3 the medullary region, and ROI 4 the main renal artery region. Subsequently, the contrast agent perfusion curve was fitted using the built-in functions to obtain the time-intensity curve. ROI was manually drawn on the B-mode image in dual-screen display mode, and the VUEBOX software automatically calculated the area of the ROI cm^2^. The software evaluates the following aspects:

1. Peak Enhancement (PE);
2. Rise time (RT);
3. Mean Transit Time (local) (mTTl);
4. Time To Peak (TTP);
5. Fall Time (FT);
6. Wash-out AUC;
7. Wash-in Rate;
8. Wash-in and Wash-out AUC.

## 4 Statistical methods

Data analysis was conducted using SPSS 26.0 software. For continuous data, normality was assessed. if the data followed a normal distribution, it was expressed as mean ± standard deviation (x ± s). One-way analysis of variance (ANOVA) was used for intergroup comparisons. If the continuous data did not follow a normal distribution, it was expressed as median (P25-P75), and intergroup comparisons were performed using the independent samples Kruskal-Wallis test. Pairwise comparisons were conducted using the Bonferroni method to adjust the significance level for multiple comparisons. A significance level of *P* < 0.05 was considered statistically significant. The diagnostic performance of parameters that exhibited differences was evaluated using ROC curves. The optimal cutoff value was determined based on the Youden index, and the sensitivity and specificity were calculated at each cutoff value.

## 5 Result

### 5.1 General data comparison

During the data collection period from July 2023 to December 2023, there were a total of 87 cases of kidney transplantation. Among them, eight cases were excluded due to transplant renal artery occlusion, and six cases due to severe perirenal fluid accumulation. Thus, 73 patients were included, comprising 47 males (63%) and 26 females (35%). The ages ranged from 22 to 62 years, with a mean age of (43.69 ± 11.02) years.

Among the 73 patients, 26 cases (36%) experienced impaired recovery of transplant renal function. Of these, 18 cases (24%) were clinically diagnosed with acute rejection, and eight cases (9%) were clinically diagnosed with delayed graft function recovery. For further analysis, these 18 patients were included in the AR group, eight patients in the DGF group, and the remaining 47 patients with good post-transplant renal function recovery were classified into the stable renal function group. Postoperative renal graft function recovery were classified into the stable renal function group.

The differences in serum creatinine levels, glomerular filtration rate, and β_2_-microglobulin among the three groups were statistically significant (all *P* < 0.05). Pairwise comparisons showed significant differences in serum creatinine levels, glomerular filtration rate, and β_2_-microglobulin between the stable renal function group and the AR group, as well as between the stable renal function group and the DGF group (*P* < 0.05). In all 25 patients in the AR and DGF groups, postoperative β_2_-microglobulin and glomerular filtration rate increased, while serum creatinine levels decreased.

The differences in age, height, and weight among the three groups were not statistically significant (*P* > 0.05), as shown in Table 1.

**Table 1:** Population demographics and laboratory parameters of the study population.

**Parameter**	**Control (*n* = 47)**	**AR (*n* = 18)**	**DGF (*n* = 8)**	**H**	**P**
Creatinine value μmol/L	170.00 (135.00,238.00)	497.00 (383.25,624.00)	652.15 (488.50,732.25)	40.053	0.000
β2-MG/L	3.78 (2.92, 5.17)	8.21 (5.80, 15.61)	11.36 (7.06, 15.76)	35.190	0.000
eGFR	42.80 (28.29, 49.39)	13.35 (9.59, 17.50)	10.80 (9.13, 11.55)	42.747	0.000
Urea nitrogen mmol/L	20.49 (16.24, 25.58)	21.38 (13.22, 35.61)	25.51 (81.84, 41.15)	2.897	0.240
Uric acid	416.00 (336.50, 529.50)	523.50 (337.00, 705.75)	467.00 (290.00, 628.25)	1.424	0.491
Urine volume ml/L	129.00 (120.00, 146.00)	196.00 (126.25, 292.75)	117.50 (53.25, 144.75)	8.990	0.011
Age	44.00 (34.00, 52.50)	45.00 (33.25, 52.75)	41.00 (35.25, 53.25)	0.014	0.993
Weight (Kg)	61.00 (53.73, 70.50)	59.83 (52.63, 74.18)	58.50 (50.68, 67.50)	0.390	0.823
Height (cm)	168.00 (160.00, 170.00)	162.00 (160.00, 170.00)	166.50 (158.50, 169.50)	0.426	0.808

Data are presented as M(QR). *P* < 0.05 is considered significant. AR, acute rejection group; DGF, delayed graft function group; gray shading indicates significant differences between groups.

### 5.2 Ultrasound

All 87 patients underwent postoperative ultrasound examinations (USD and CEUS). Ultrasound was used to gather information about size and position. There were no significant differences observed in ultrasound features among the three groups, including the length, width, and height of the transplanted kidney, as well as cortical thickness and medullary size. Doppler ultrasound was used to measure peak systolic velocity (PSV) and resistive index (RI) at different arterial levels, as well as to visualize the arcuate arteries and interlobar arteries. Comparison of renal segmental artery resistive index among the three groups of patients showed statistical significance (*P* < 0.05). Both the AR group and the DGF group exhibited higher renal segmental artery RI compared to the stable renal function control group. Please refer to Table 2 for details.

**Table 2:** Ultrasound parameters of the study population.

**Parameter**	**Control (*n* = 47)**	**AR (*n* = 18)**	**DGF (*n* = 8)**	**Statistic**	**P**
Length	105.98 ± 11.51	106.39 ± 10.70	103.29 ± 8.04	0.15	0.86
Width	45.64 ± 6.08	45.61 ± 5.79	46.57 ± 6.16	0.21	0.81
Thickness	43.73 ± 5.50	43.89 ± 6.52	45.14 ± 6.79	0.05	0.96
Renal cortical thickness mm	6.33 ± 1.40	6.61 ± 1.42	6.00 ± 1.63	0.30	0.74
Pyramid size of kidney (mm)	181.64 ± 94.30	165.11 ± 71.11	151.29 ± 42.07	0.24	0.79
Arcuate arteries PSV	33.25 ± 11.94	28.61 ± 12.13	28.00 ± 16.74	0.91	0.41
Renal arcuate arteries; RI	0.60 ± 0.07	0.64 ± 0.09	0.59 ± 0.10	1.38	0.26
Interlobar arteries PSV	30.32 ± 10.19	29.22 ± 9.30	25.00 ± 7.85	0.14	0.87
Interlobar arteries; RI	0.62 ± 0.07	0.67 ± 0.09	0.63 ± 0.08	2.17	0.12
Segmental artery PSV	45.98 ± 15.83	49.00 ± 11.79	47.57 ± 23.75	0.25	0.78
**Segmental artery; RI**	0.61 ± 0.09	0.68 ± 0.09	0.63 ± 0.11	3.20	0.04
Renal artery PSV	70.56 ± 30.50	60.44 ± 31.89	56.57 ± 27.81	1.35	0.27
Renal artery; RI	0.67 ± 0.09	0.69 ± 0.08	0.67 ± 0.08	0.46	0.63

Data are presented as mean ± SD. *P* < 0.05 is significant.

### 5.3 Contrast-enhanced ultrasound

Contrast-enhanced ultrasound (CEUS) identified the cortex, medulla, subcapsular area, and main renal artery as four regions of interest (ROIs), each region having 12 variables. A comparative analysis of quantitative perfusion parameters of CEUS was conducted among the three groups. When compared to the AR group, statistically significant differences (*P* < 0.05) were observed in cortex time-to-peak (TTP), medulla TTP, main renal artery rise time (RT), main renal artery TTP, and main renal artery fall time (FT) in the DGF group, while other parameters showed no statistical differences (*P* > 0.05). In the cortex ROI, the DGF group showed longer TTP compared to the AR group. In the medulla ROI, the DGF group exhibited longer RT and TTP compared to the AR group. In the main renal artery ROI, the DGF group showed longer TTP, RT, and FT compared to the AR group. When compared to the stable renal function group, statistically significant differences (*P* < 0.05) were observed in cortex TTP, main renal artery RT, main renal artery TTP, and main renal artery FT in the DGF group, while other parameters showed no statistical differences (*P* > 0.05). In the main renal artery ROI, the DGF group showed longer TTP, RT, and FT compared to the group with stable renal function. Please refer to Table 3 for details.

**Table 3:** Contrast-enhanced ultrasound parameters of the study population.

**Parameter**	**Control (*n* = 47)**	**AR group (*n* = 18)**	**DGF group (*n* = 8)**	**H/F**	**P**
**Large subcapsular ROI**					
MeanLin [a. u],	5,854.93 (3,030.07, 14,189.66)	5,928.04 (3,280.64, 10,350.53)	6,341.06 (4,890.04, 10,687.38)	9.417	0.833
PE, AU	15,809.29 (6,809.78, 42,053.21)	12,853.93 (5,277.97, 26,028.36)	13,436.54 (6,892.55, 16,950.17)	1.383	0.501
WiAUC, AU	78,895.77 ± 139,881.65	4,2699.21 ± 40,826.12	44,772.35 ± 18,843.83	0.797	0.455
Rise time, s	3.57 (2.53, 4.67)	3.82 (3.24, 5.49)	6.08 (3.79, 6.84)	5.902	0.052
mTTl, s	31.98 ± 36.81	29.19 ± 26.33	61.31 ± 47.04	2.562	0.844
TTP, s	4.93 (3.60, 6.68)	4.82 (4.07, 7.59)	7.96 (5.14, 8.81)	5.209	0.074
WiR, AU	6,266.72 (2,911.49, 18,680.42)	5,719.51 (1,895.68, 13,955.58)	3,454.69 (1,598.29, 5,929.72)	3.287	0.193
WoAUC, AU	82,014.49 (44,840.22, 179,601.05)	66,167.90 (25,054.51, 151,276.13)	118,868.48 (65,565.42, 138,493.65)	1.563	0.458
WiWoAUC, AU	116,894.97 (64,027.18, 263,082.50)	94,533.74 (40,283.99, 220,992.48)	172,323.42 (93,818.80, 198,523.52)	1.580	0.454
Fall time, s	10.79 ± 7.88	10.70 ± 5.58	14.52 ± 6.51	0.960	0.388
WoR, AU	1,964.55 (733.25, 6,428.44)	1,549.66 (448.83, 3,325.38)	1,218.35 (369.71, 1,904.68)	1.993	0.369
QOF, %	78.75 (60.26, 86.10)	70.32 (41.85, 83.25)	62.81 (38.75, 79.66)	3.605	0.165
Area, cm^2^	0.34 (0.18, 0.46)	0.21 (0.12, 0.48)	0.21 (0.13, 0.32)	6.109	0.047
**Cortex ROI**					
MeanLin, AU	6,253.17 (3,528.55, 14,884.91)	5,704.62 (3,686.72, 11,616.19)	7,429.82 (4,346.69, 10,787.41)	0.154	0.926
PE, AU	21,676.93 (8,800.95, 54,146.36)	19,540.89 (7,891.65, 29,793.47)	17,738.30 (9,510.82, 29,216.89)	0.848	0.654
WiAUC, AU	86,967.85 ± 134,851.39	58,373.22 ± 78,998.49	40,250.84 ± 19,109.76	0.791	0.457
Rise time, s	2.69 (2.27, 3.48)	2.81 (2.10, 3.05)	3.56 (2.43, 5.19)	3.539	0.170
mTTl, s	9.85 (5.09, 17.63)	9.42 (5.34, 21.78)	21.96 (12.73, 48.46)	5.117	0.077
**TTP, s**	3.74 (2.97, 4.81)	3.82 (3.36, 4.19)	6.00 (4.28, 8.06)	9.417	0.009
WiR, AU	25,644.78 ± 32,879.86	40,981.19 ± 119,078.92	9,593.35 ± 8,398.91	0.721	0.490
WoAUC, AU	257,961.07 ± 644,684.12	108,230.99 ± 122,048.45	80,172.16 ± 41,726.48	0.766	0.469
WiWoAUC, AU	344,928.92 ± 768,717.32	166,604.21 ± 200,132.11	120,422.99 ± 58,091.09	0.794	0.456
Fall time, s	7.92 ± 5.53	5.85 ± 2.11	8.28 ± 4.34	1.346	0.267
WoR, AU	7,156.53 ± 8,981.41	20,216.27 ± 66,950.18	4,048.59 ± 3,970.57	1.112	0.335
QOF, %	68.41 (52.50, 73.76)	66.62 (64.01, 74.33)	58.90 (45.54, 74.98)	1.814	0.404
Area, cm^2^	0.05 (0.04, 0.06)	0.05 (0.03, 0.06)	0.04 (0.03, 0.07)	1.055	0.590
**Medulla ROI**					
MeanLin [a. u],	2,156.19 (1,196.42, 3,533.35)	2,203.81 (937.91, 3,062.92)	1,440.14 (1,178.15, 4,299.08)	0.439	0.803
PE, AU	4,725.32 (2,379.01, 7,480.09)	4,517.33 (1,692.25, 7,653.59)	3,717.89 (2,073.53, 11,033.82)	0.285	0.867
WiAUC, AU	18,230.20 (11,282.23, 35,798.65)	13,173.62 (9,331.63, 28,902.81)	18,861.08 (9,687.29, 29,414.19)	2.258	0.323
Rise time, s	8.48 ± 5.30	6.76 ± 3.27	7.42 ± 3.66	0.913	0.406
mTTl, s	34.13 (22.55, 62.75)	34.63 (18.21, 44.30)	43.42 (12.54, 54.75)	1.059	0.589
**TTP, s**	10.86 (7.44, 18.81)	10.89 (6.89, 15.05)	19.85 (18.08, 23.27)	8.840	0.012
WiR, AU	916.03 (345.83, 1,593.99)	992.26 (423.85, 2,300.68)	607.30 (325.18, 3,236.26)	0.152	0.927
WoAUC, AU	68,887.99 ± 93,170.07	37,002.87 ± 27,302.62	46,975.94 ± 44,707.31	1.187	0.311
WiWoAUC, AU	97,409.13 ± 112,782.46	56,130.45 ± 39,715.61	69,114.48 ± 59,344.96	1.331	0.271
Fall time, s	12.42 (8.45, 27.65)	12.07 (7.14, 22.43)	12.47 (6.92, 25.42)	1.640	0.44
WoR, AU	369.53 (119.25, 669.62)	397.79 (99.88, 1,059.28)	379.80 (109.60, 1,014.00)	0.215	0.898
QOF, %	58.24 (38.18, 63.86)	57.60 (32.24, 65.84)	52.01 (36.35, 60.83)	0.394	0.821
Area, cm^2^	0.05 (0.04, 0.06)	0.04 (0.03, 0.05)	0.04 (0.02, 0.05)	2.257	0.324
**Renal artery ROI**					
MeanLin [a. u],	3,628.08 (1,401.23, 10,342.50)	4,188.97 (605.16, 8,185.81)	3,511.82 (1,377.50, 6,751.11)	0.421	0.810
PE, AU	16,343.01 (5,873.17, 41,971.16)	10,834.76 (1,084.96, 43,973.05)	8,486.42 (3,210.88, 15,465.05)	2.807	0.246
WiAUC, AU	19,952.70 (7,753.73, 60,651.36)	14,827.31 (1,587.47, 56,428.05)	26,240.56 (8,599.84, 39,551.82)	1.090	0.580
**Rise time, s**	2.01 (1.63, 3.34)	2.12 (1.57, 2.73)	3.67 (1.63, 3.34)	10.284	0.006
mTTl, s	18.91 ± 28.99	17.71 ± 31.62	61.34 ± 58.41	5.742	0.005
**TTP, s**	2.86 (2.25, 4.63)	3.10 (2.48, 3.65)	4.77 (4.13, 6.89)	9.272	0.010
WiR, AU	83,867.78 ± 185,880.65	20,493.72 ± 31,098.59	20,858.05 ± 50,701.95	1.444	0.243
WoAUC, AU	45,697.37 (14,950.61, 175,209.66)	31,894.19 (3,302.54, 107,573.04)	48,769.32 (20,087.38, 97,599.52)	1.221	0.543
WiWoAUC, AU	46,994.97 (5,546.44, 140,993.77)	75,009.88 (33,731.97, 131,549.00)	4.77 (3.21, 7.85)	1.156	0.561
**Fall time, s**	4.73 (3.17, 7.91)	4.99 (3.26, 6.84)	9.68 (7.05, 15.43)	8.516	0.014
WoR, AU	4,062.75 (1,144.56, 15,989.26)	2,413.39 (250.94, 6,795.94)	892.02 (301.74, 2,111.83)	4.329	0.115
QOF, %	47.51 (35.30, 66.36)	53.34 (34.60, 68.12)	47.89 (36.82, 67.11)	0.120	0.942
Area, cm^2^	0.04 (0.03, 0.07)	0.04 (0.03, 0.06)	0.05 (0.05, 0.07)	2.975	0.226

Data are presented as mean ± SD or M(QR). *P* < 0.05 is significant. DGF, delayed graft function; AR, acute rejection; AU, arbitrary units; AUC, area under the curve; MeanLin, mean linearized signal intensity; mTTl, mean transit time (local); PE, peak enhancement; TTP, time to peak; R, rate; PI, perfusion index; QOF, quality of fit; Wi, wash-in; and Wo, wash-out.

**Figure 1:**
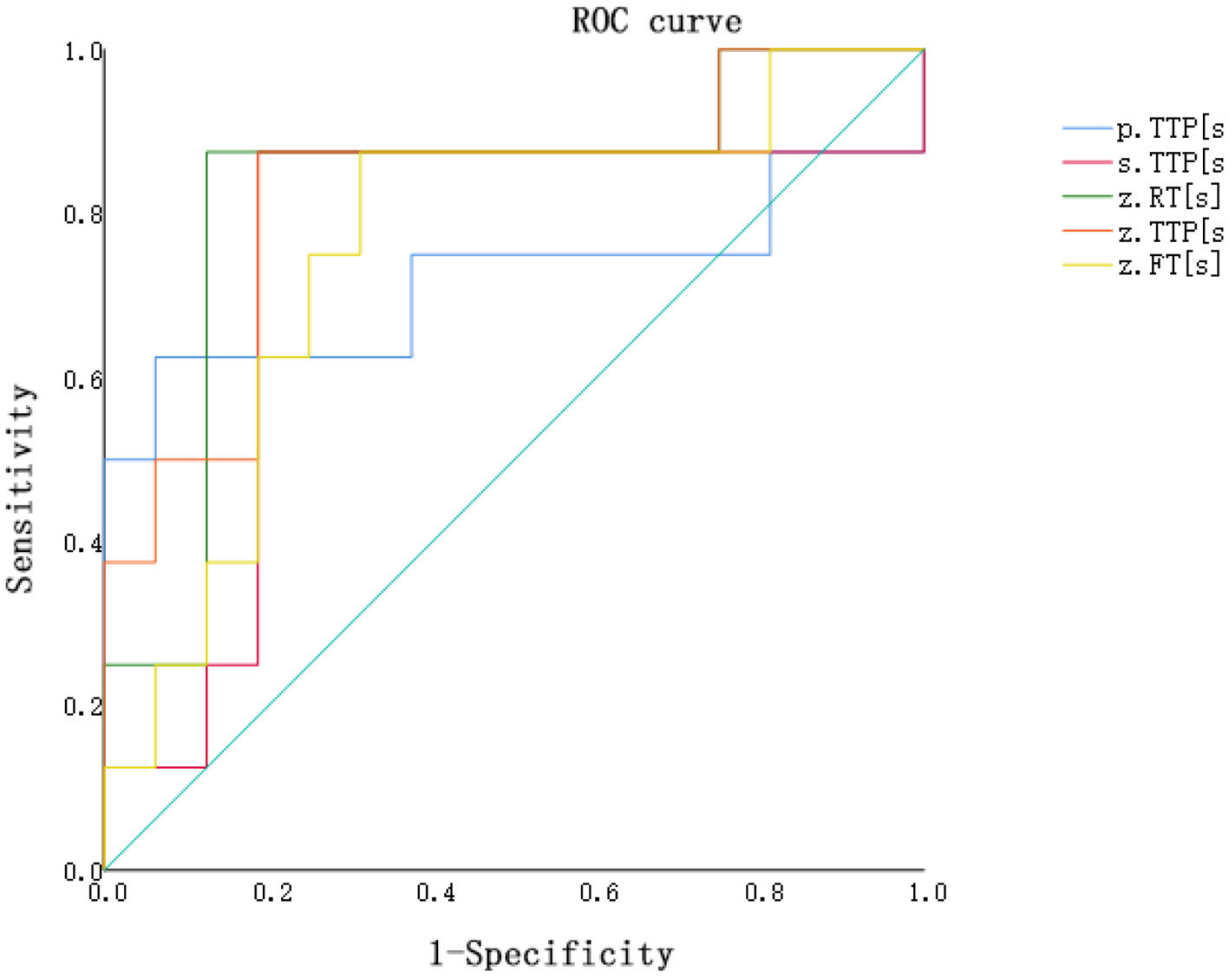
ROC curve for distinguishing between the AR group and the DGF group based on contrast-enhanced ultrasound perfusion parameters. P.TTP, cortex TTP; S.TTP, medulla TTP; Z.RT, renal artery RT; Z.TTP, renal artery TTP; Z.FT, renal artery FT.

Contrast-enhanced ultrasound (CEUS) for diagnosing acute rejection of transplanted kidneys revealed the accuracy in distinguishing between DGF and AR as follows: Renal artery TTP = Renal artery RT > Renal artery FT > Medulla TTP > Cortex TTP (with areas under the curve of 0.828, 0.828, 0.758, 0.742, and 0.719, respectively). Please refer to Table 4 and Figure 1 for details.

**Table 4:** Diagnostic performance of the parameters.

**Parameter**			**Area under the curve**	**95% confidence interval**	**Cutoff**	**Sensitivity %**	**Specificity %**
**AR vs. DGF**							
Medulla TTP			0.742	0.446–0.991	0.687	0.875	0.812
Cortex TTP			0.719	0.499–0.985	0.562	0.625	0.937
Renal artery RT			0.828	0.634–1.000	0.75	0.875	0.875
Renal artery FT			0.758	0.546–0.970	0.562	0.875	0.687
Renal arteryTTP			0.828	0.638–1.000	0.687	0.875	0.812

## 6 Discussion

Conventional ultrasound offers the advantages of being simple, real-time, and feasible for bedside examinations, making it the preferred modality for post-transplant kidney monitoring. It provides information on the overall morphology and blood flow perfusion of the transplanted kidney by observing its tissue structure and arterial and venous blood flow. Currently, peak systolic velocity (PSV) and resistive index (RI) of the renal arteries are widely recognized as two commonly used parameters for post-transplant kidney monitoring (9).

This study did not find any morphological conventional ultrasound parameters capable of diagnosing acute rejection or delayed function recovery in transplanted kidneys. Regarding Doppler parameters, we observed a higher resistive index (RI) in the renal segmental arteries of the acute rejection group (*P* < 0.05), indicating a statistically significant difference. However, there were no significant differences observed in the RI of the interlobar arteries, arcuate arteries, and main renal arteries. This suggests that RI may not serve as a reliable marker for diagnosing functional impairment in transplanted kidneys (10). This conclusion is consistent with the findings of some scholars, such as the study by Goyal et al. in 2020, which found no significant difference in the resistive index (RI) between the stable renal function group and the group with renal functional impairment (8). According to the calculation formula of the resistive index (RI), it represents the percentage decrease in blood flow during diastole relative to peak systole in renal vasculature, providing a quantitative measure of renal microcirculation (11). However, the increase in RI is not solely attributable to changes in microcirculation, as RI is influenced by factors such as vascular resistance, pulse pressure, heart rate, and rhythm (12). Moreover, according to the principles of Doppler imaging, the detection of RI is susceptible to the angle between the sound beam and the direction of blood flow. Hence, there is still controversy surrounding the application of RI in the ultrasound diagnosis of renal transplantation.

Contrast-enhanced ultrasound examination (CEUS) is a diagnostic method that utilizes the principle of backscatter of microbubble contrast agents combined with ultrasound nonlinear imaging technology. This technique enhances the resolution of ultrasound imaging and increases diagnostic sensitivity and specificity (13). Microbubble contrast agents are vascular tracers similar to red blood cells. Their blood flow imaging is not affected by the direction of blood flow or the angle of the ultrasound beam. The sensitivity of blood flow detection with microbubble contrast agents is significantly higher than that with color Doppler imaging. Under consistent acoustic window conditions, they theoretically overcome cross-sectional and individual differences, thereby significantly enhancing the detection capability of low flow and low velocity.

The pathogenesis of delayed graft function (DGF) remains unclear to date. It is often considered to be caused by repeated ischemia-reperfusion injury to the renal tubules (14, 15). Early diagnosis and timely treatment of DGF are crucial for the long-term survival of the transplanted kidney. Currently, the diagnosis and differential diagnosis of DGF rely on histopathological biopsy, which is an invasive procedure. Due to the psychological stress associated with this procedure, some transplant patients refuse it. Therefore, improving the accuracy of early DGF diagnosis is of great importance. Clinically, several methods have been proposed to further accurately diagnose DGF, such as observing postoperative anuria and oliguria, monitoring SCr levels, and assessing whether patients require dialysis based on clinical judgment. However, these methods have certain limitations influenced by early postoperative fluid management, treatment plans, insurance policies, and varying interpretations of dialysis indications by different physicians, all of which affect the timing of dialysis initiation. This study found that the cortical and medullary TTP and the RT, FT, and TTP of the main renal artery in the DGF group were longer than those in the AR group. This provides new focal points for clinicians in the early diagnosis of DGF and the differentiation between early DGF and AR.

Approximately 95% of renal blood flow originates from the renal cortex (16). Complications occurring after renal transplantation may affect microcirculation, leading to a disproportionate decrease in medullary blood flow relative to total renal blood flow (17).

**Figure 2:**
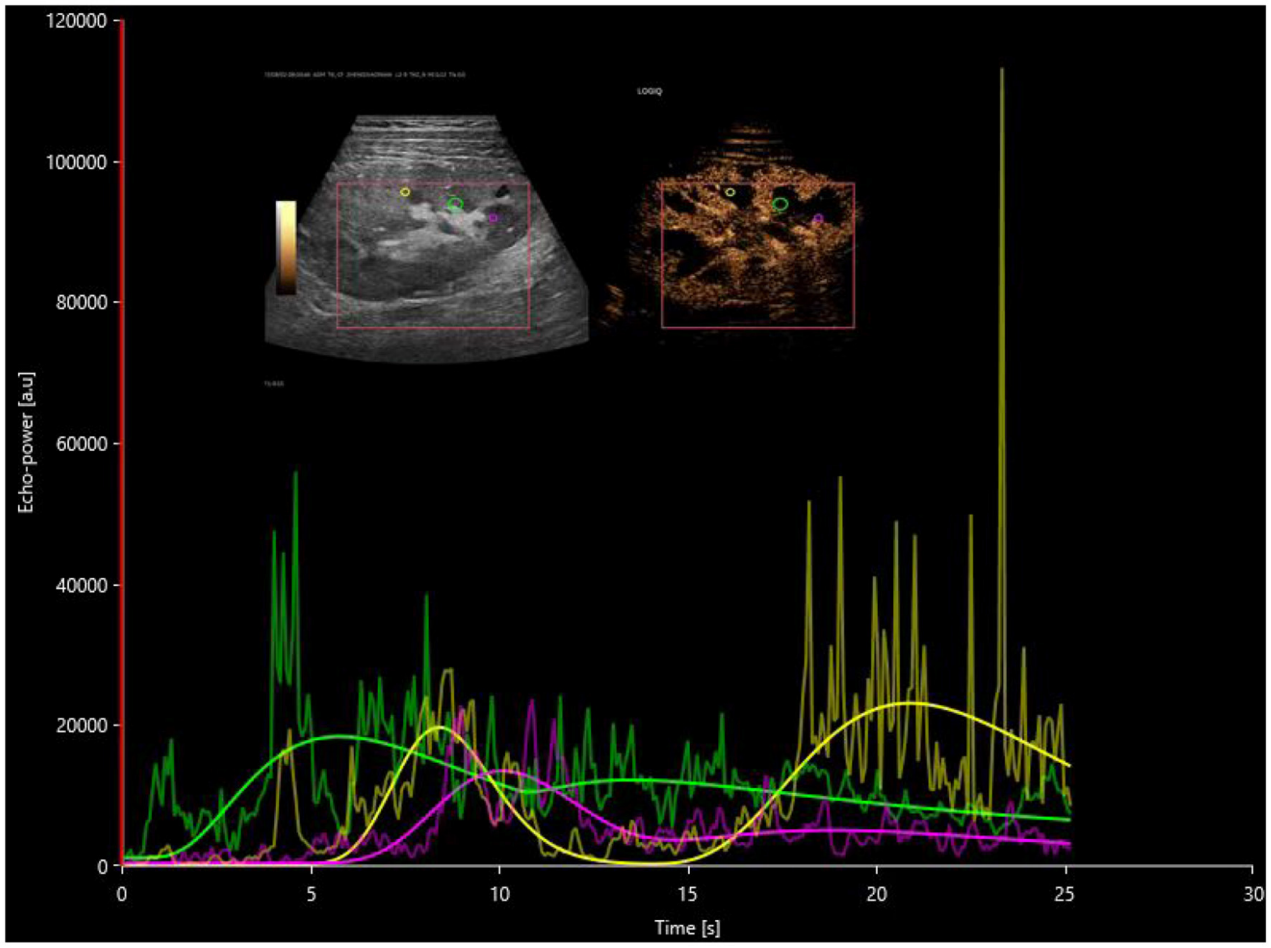
Postoperative time-intensity curves of contrast-enhanced ultrasound in the DGF group, with regions of interest (ROIs) highlighted for the cortex (yellow), medulla (pink), and a combination of partial cortex and medulla under the large capsule (green).

**Figure 3:**
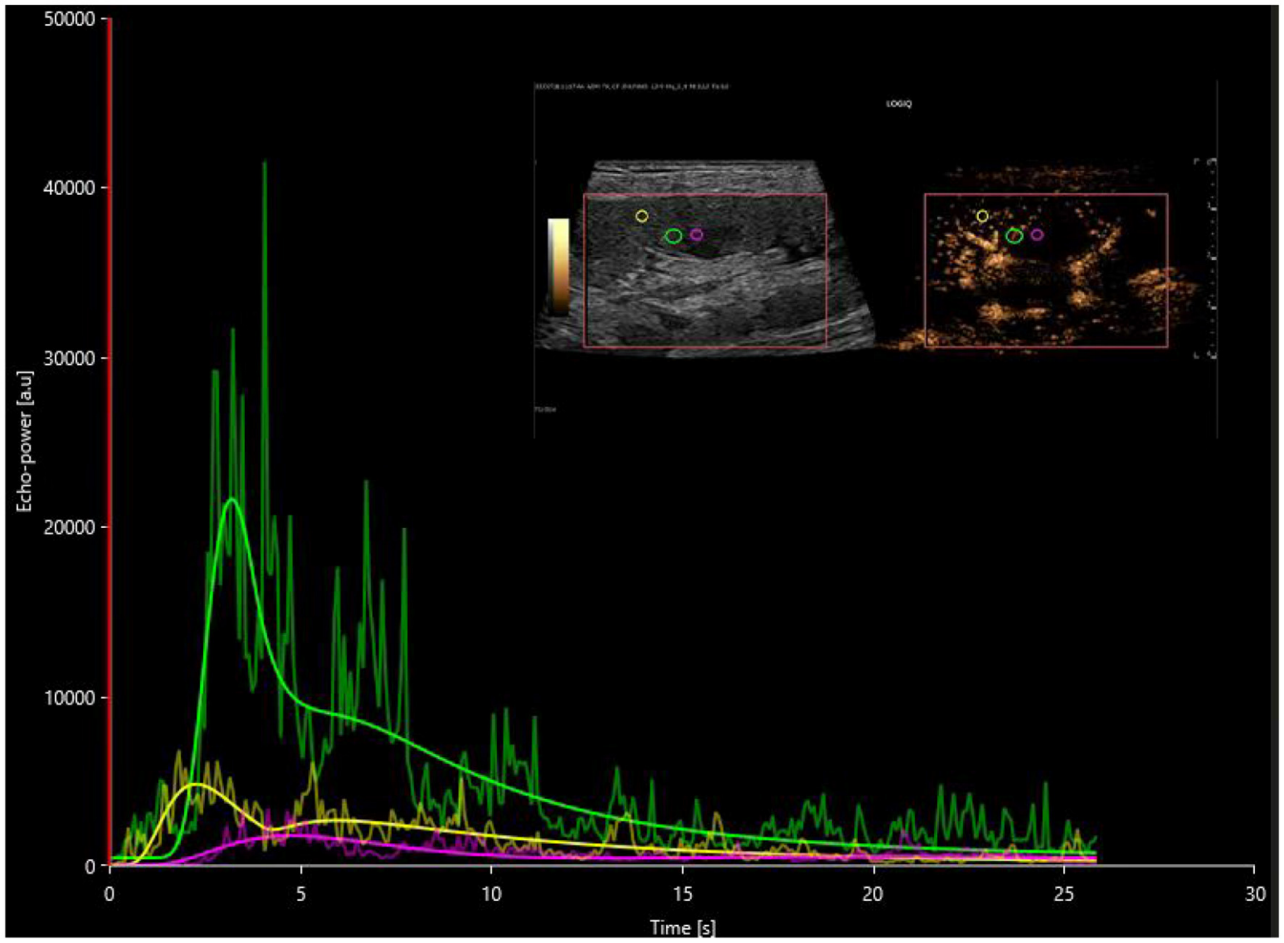
Postoperative time-intensity curves of contrast-enhanced ultrasound in the AR group, with regions of interest (ROIs) highlighted for the cortex (yellow), medulla (pink), and a combination of partial cortex and medulla under the large capsule (green).

The investigation utilized contrast-enhanced ultrasound along with VUEBOX quantitative analysis software to determine that the time to peak (TTP) in both the cortical and medullary regions of interest showed prolonged durations in the DGF cohort compared to both the stable renal function cohort and the AR cohort, indicating statistically significant differences (*P* < 0.05). These findings can be employed to distinguish between DGF and AR, as the TTP in the cortical and medullary regions in the DGF group was significantly extended compared to the AR group, as illustrated in Figures 2, 3. The reduced rate of enhancement of the contrast agent within the transplanted kidney suggests that delayed recovery of renal function may be due to increased microcirculatory resistance and uneven distribution of resistance, leading to significant delays in the inflow of the contrast agent (18). This finding aligns with previous studies. Moreover, this study identified four distinct regions of interest and found that different regions reflected varying perfusion conditions within the transplanted kidney. Nonetheless, parameters within the cortical and medullary regions seem to better capture differences in microcirculatory blood flow perfusion within the transplanted kidney. This suggests that during contrast-enhanced ultrasound examination, increased resistance hinders the entry of the contrast agent from the cortex into the medulla in the transplanted kidneys of the DGF cohort, consistent with its pathological changes (19, 20).

**Figure 4:**
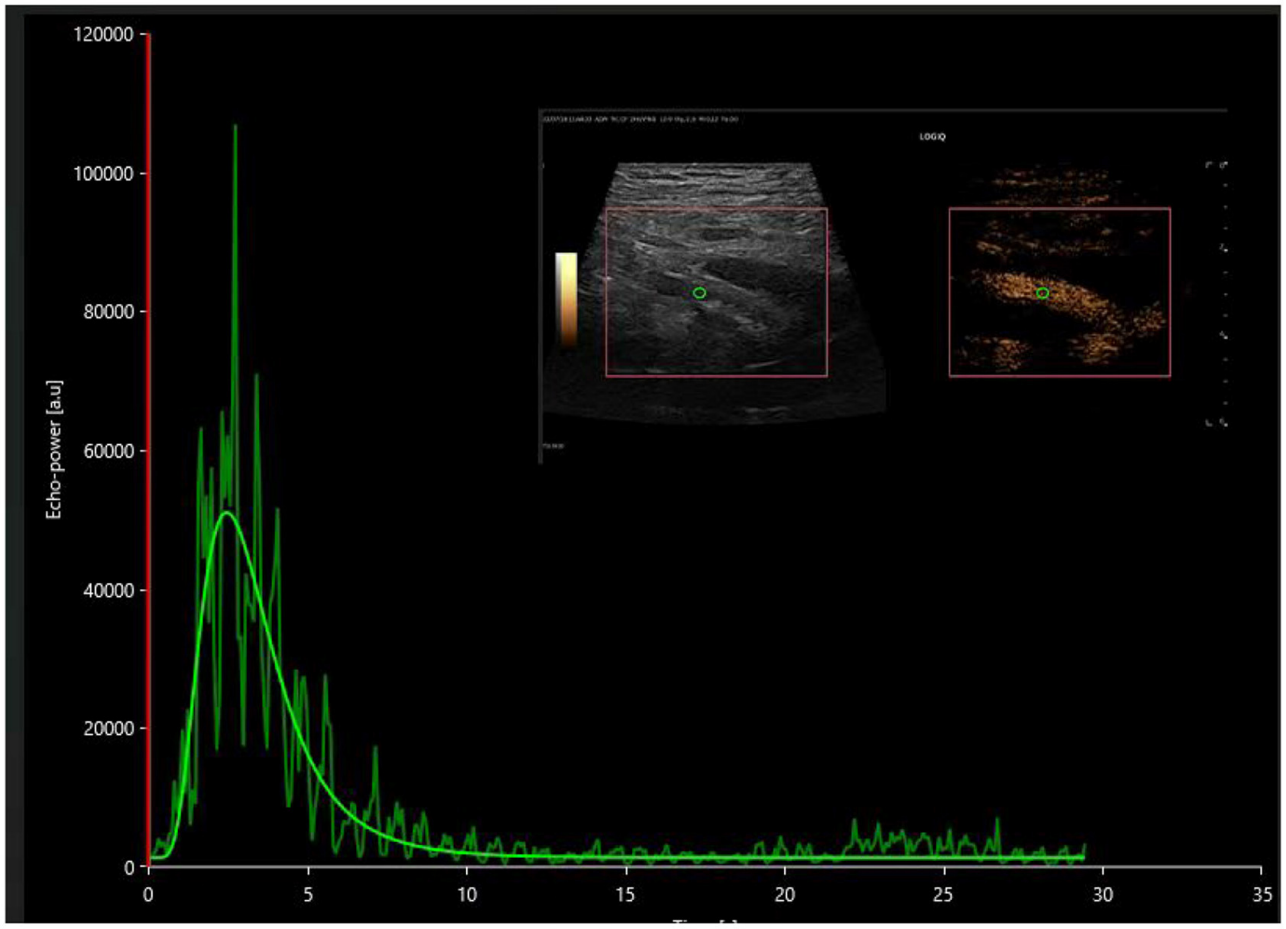
The time-intensity curve of the main renal artery on contrast-enhanced ultrasound in the postoperative acute rejection (AR) group.

**Figure 5:**
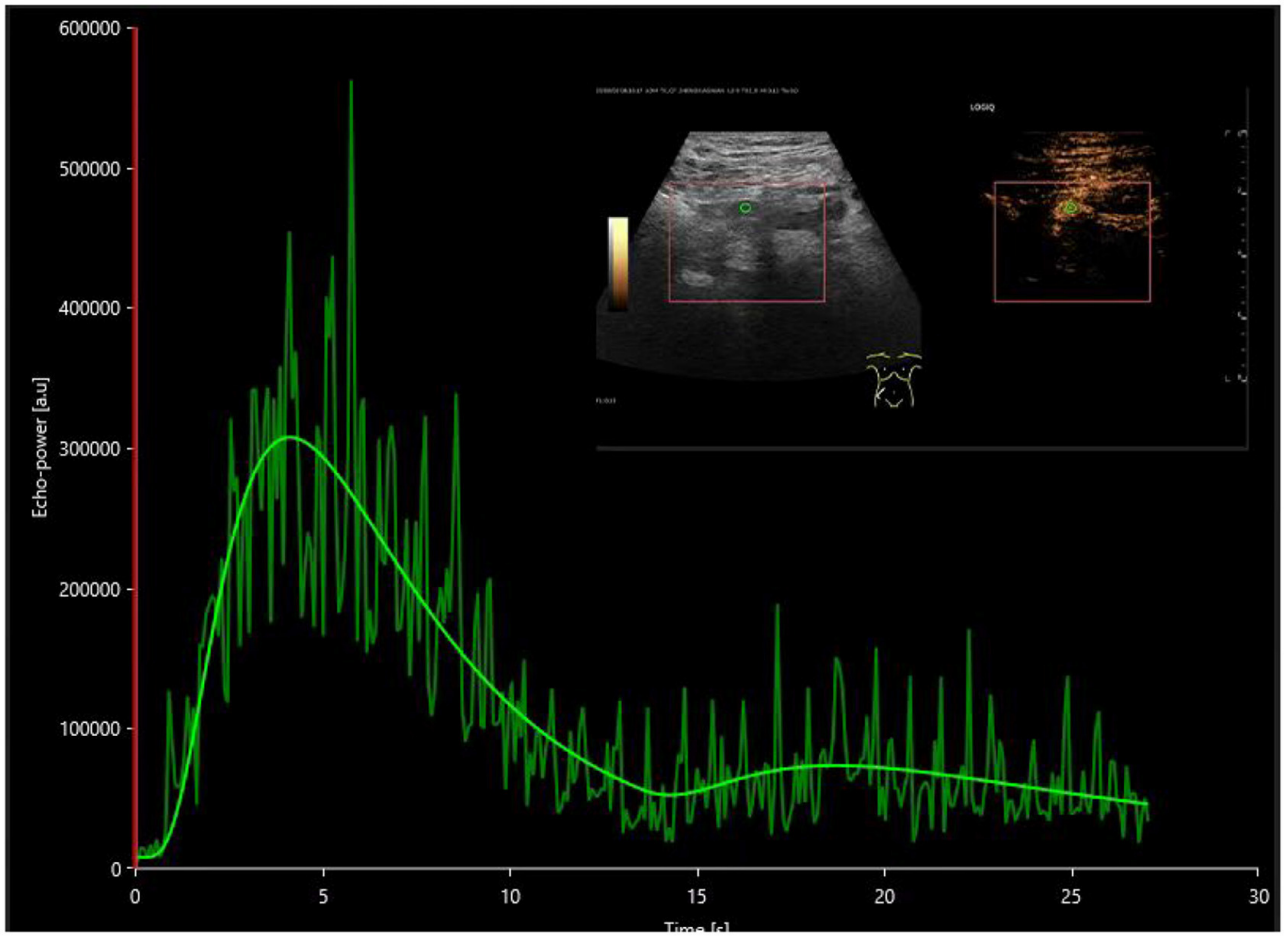
The time-intensity curve of the main renal artery on contrast-enhanced ultrasound in the postoperative DGF group.

In this investigation, all renal transplant procedures were executed utilizing an end-to-side anastomosis technique, with complications pertinent to renal vasculature arising from transplantation surgery being systematically excluded. In patients exhibiting stable recovery of renal function, contrast-enhanced ultrasound unveiled a sequential enhancement pattern, progressing from the iliac artery to the main renal artery, interlobar artery, interlobular artery, and arcuate artery. Employing the perfusion pattern of the main renal artery in the AR group, as illustrated in Figure 4, as a reference, we analyzed the perfusion pattern of the main renal artery in the DGF group, as depicted in Figure 5.

This study first discovered significant differences in CUES parameters in the region of interest of the main renal artery in transplant kidneys. All renal transplant procedures were performed using an end-to-side anastomosis technique, and complications related to renal vasculature caused by the transplantation surgery were excluded. In patients with stable recovery of renal function, contrast-enhanced ultrasound revealed sequential enhancement of the iliac artery, main renal artery, interlobar artery, interlobular artery, and arcuate artery. In the cohort experiencing delayed graft function (DGF), the sequence of contrast enhancement within the transplanted kidney mirrored that observed in the stable renal function cohort, albeit with a notably diminished rate of enhancement. The presence of renal vascular resistance during renal dysfunction may lead to the cessation or even reversal of blood flow within the renal artery and interlobar artery during diastole (21, 22).

This investigation observed a higher renal artery resistance index (RI) in the DGF group using two-dimensional ultrasound, even in the absence of diastolic reflux. Such observations could potentially be attributed to the limited sample size and possible errors introduced by variations in patient heart rate, blood pressure, and angles. Nonetheless, contrast-enhanced ultrasound imaging of the main renal artery and subsequent quantitative analysis using VUEBOX software revealed a statistically significant prolongation in time-to-peak (TTP), rise time (RT), and fall time (FT) within the main renal artery in the DGF group compared to both the stable renal function and acute rejection cohorts. This difference may be due to pathological changes in patients with delayed graft function (DGF), such as detachment of tubular epithelial cells, renal enlargement, and increased renal tension, leading to increased microvascular resistance.

Contrast-enhanced ultrasound shows promising potential in distinguishing acute rejection and delayed graft function (DGF) in renal transplantation. A study conducted by Grzelak et al. in 2011 substantiated this potential (18). In comparison to the stable renal function cohort, the time taken for the contrast agent to reach the cortex and medulla of the transplanted kidney was significantly prolonged in the DGF cohort. This finding was corroborated by a study conducted by Liang et al. (23). By categorizing 44 patients into NGF and DGF cohorts, the ultrasonic contrast study revealed that, compared to the NGF cohort, microcirculation perfusion within the transplanted kidneys of the DGF cohort showed increased levels, earlier arrival times, shorter times to peak enhancement, and higher peak enhancement intensity. These findings suggest that DGF may lead to increased microcirculatory abnormalities in transplanted kidneys, possibly associated with inflammatory responses triggered by DGF, while the reduced time to peak enhancement may be connected to the expansion of small to medium-sized arteries (24).

Contrast-enhanced ultrasound (CEUS) enables real-time dynamic observation of the enhancement pattern in transplanted kidneys. Conventional quantitative analysis, however, is susceptible to the influence of analysis software and ultrasound machine models, leading to significant variations in parameter values obtained from different machines. Nevertheless, external quantitative perfusion software such as VUEBOX can be applied to most ultrasound machine models to perform motion calibration, thereby reducing errors caused by patient respiratory motion. Although this study had a relatively small sample size due to strict inclusion criteria during the contrast imaging process, the VUEBOX quantitative analysis software provided more objective diagnostic data for the occurrence of DGF and acute rejection reactions following renal transplantation. However, this study was conducted at a single center. Further collaboration across multiple centers is warranted to expand the sample size and utilize more standardized pathological gold standards to validate the results of renal transplant CEUS quantitative analysis more comprehensively.

However, this study has several limitations. Being a single-center study, there is a need for further multi-center collaboration to increase the sample size and employ more standardized pathological gold standards to further validate the results of CEUS quantitative analysis in transplant kidneys. The study did not consider pre-transplant primary conditions such as diabetic nephropathy leading to renal failure or IgA nephropathy leading to renal failure, nor did it explore the quantitative differences in ultrasound contrast for these conditions post-transplant. Additionally, this study only investigated the diagnostic utility of two-dimensional ultrasound and contrast-enhanced ultrasound for post-transplant kidneys, without incorporating other imaging techniques such as elastography, MVI, or B-flow.

In conclusion, contrast-enhanced ultrasound combined with VUEBOX quantitative analysis software provides an intuitive, non-invasive method for detecting and differentiating post-transplant DGF and acute rejection. This approach offers more objective diagnostic data and can compensate for the limitations of two-dimensional ultrasound, showing good clinical application value. However, further research is needed to verify its accuracy and reliability.

## Data Availability

The original contributions presented in the study are included in the article/Supplementary material, further inquiries can be directed to the corresponding authors.
